# Contribution of amniotic membrane to the healing of iatrogenic vas deferens injury

**DOI:** 10.3906/sag-2012-287

**Published:** 2021-06-28

**Authors:** Sabri DEMİR, Ahmet ERTÜRK, Mehmet ZENGİN, Dinçer YILDIZ, Siyami KARAHAN, Emrah ŞENEL

**Affiliations:** 1 Department of Pediatric Surgery, Faculty of Medicine, KırıkkaleUniversity, Kırıkkale Turkey; 2 Department of Pediatric Surgery, Children Hospital, Ankara City Hospital, Ankara Turkey; 3 Department of Pathology, Faculty of Medicine, KırıkkaleUniversity, Kırıkkale Turkey; 4 Department of Anatomy, Faculty of Veterinary, Kırıkkale University, Kırıkkale Turkey; 5 Department of Hystology, Faculty of Veterinary, Kırıkkale University, Kırıkkale Turkey; 6 Department of Pediatric Surgery, Faculty of Medicine, Yıldırım Beyazıt University, Ankara Turkey

**Keywords:** Amniotic membrane, anastomosis, inguinal hernia, vas deferens, vasovasostomy

## Abstract

**Background/aim:**

Iatrogenic vas deferens injury is one of the most serious complications of operations in the inguinal region. Vasovasostomy is performed as treatment. However, stenosis is common after vasovasostomy. Oligospermia or azoospermia may develop and result in infertility. This study aimed to investigate the effect of amniotic membrane on healing in vas deferens injuries.

**Materials and methods:**

Four groups consisting of 10 rats each were formed. No procedure was performed in Group-I. In Group-II, the left vas deferens was transected and left to spontaneous healing. In Group-III, the left vas deferens was transected, and end-to-end anastomosis was performed. In Group-IV, the left vas deferens was transected, end-to-end anastomosis was performed, and it was closed with a wrapping of amniotic membrane on the anastomosis line. Rats were sacrificed after 60 days, and each left vas deferens was evaluated. Lumen patency was checked by passing methylene blue through the vas deferens. Subsequently, the vas deferens was evaluated both macroscopically and histopathologically. Data were evaluated using SPSS version 21.0. p < 0.05 was considered statistically significant for all variables.

**Results:**

The anastomosis lines in Group-IV healed better than those in Group-III, and less stenosis was observed. There were differences between the groups in terms of luminal patency (p = 0.009), adhesions to surrounding tissues (p = 0.02) and separation of the ends of the vas deferens (p = 0.03).

**Conclusion:**

We observed improvement on luminal patency and histology of rat vas deferens injury after surrounding human amniotic membrane on the transected and repaired surface. Further studies are needed to apply this promising result on human beings.

## 1. Introduction

Inguinal hernia repair and other inguinal region operations are the most common operations performed by pediatric surgeons [1]. Iatrogenic vas deferens injuries (IVDI) are one of the most serious complications of these operations [2]. IVDI may develop as a result of direct transection or excessive compression of the vas deferens (VD). Regardless of the mechanism, oligospermia or azoospermia may develop and result in infertility in these patients in later years [3]. The incidence of IVDI during inguinal hernia repair in children has been reported to be 0.05%–1.6% [2,4]. The frequency of VD obstruction in subfertile patients with a history of inguinal hernia repair in childhood reaches 26.7% [5]. If a surgeon recognizes unintentional transection of the VD during surgery, they may try to repair it; however, since most hospitals have neither the necessary infrastructure nor surgeons experienced in microsurgery, normal vasovasostomy is usually performed. Obstruction is often observed after vasovasostomy is performed in this way.

Human amniotic membrane (HAM) is a semipermeable, semitransparent membrane with a thickness ranging from 0.02 to 0.05 mm. It does not contain muscles, blood vessels, lymph vessels or nerve tissue [6]. HAM consists of three layers: the epithelial layer, a thick basement membrane and a stromal layer [7]. It originates from the epiblast and contains two cell types from different embryological origins: human amnion epithelial cells (HAECs) and human amnion mesenchymal stromal cells [8]. These cells secrete extracellular matrix proteins, growth factors and cytokines and have self-renewal and differentiation properties [9]. HAECs also express surface markers associated with human embryonic stem cells (HESCs), including SSEA-3, SSEA-4, TRA-1-60 and TRA-1-81, and pluripotent stem cell-specific transcription factors, such as Oct-4 and Nanog [6]. In addition, HAECs express mesenchymal stem cell (MSC) specific markers CD44, CD73, CD29, CD105 and CD90 [10].

The aim of this study was to investigate the contribution of HAM to healing in transected VD in rats.

## 2. Materials and methods

### 2.1. Creating of groups

Forty male Wistar albino rats weighing between 250 g and 350 g were used in this study. Ethics approval was obtained from the local ethics committee. Four groups, each consisting of ten rats, were created (Table 1).

**Table 1:** Forming the groups of rats.

Group #	Explanation of the groups	Number
Group-I	Control group	10
Group-II	Sham group	10
Group-III	Vas deferens transected and anastomosed only	10
Group-IV	Vas deferens transected, anastomosed and wrapped with amniotic membrane	10
	Total	40

1. Group-I (control group): No procedure was performed on these rats.

2. Group-II: The left VD was transected as described below and allowed to heal spontaneously.

3. Group-III: The left VD was transected and left to heal after performing vasovasostomy by end-to-end anastomosis.

4. Group-IV: The left VD was transected, vasovasostomy was performed, and the anastomosis line was wrapped in HAM.

### 2.2. Preparing of human amniotic membrane

**Figure 1:**
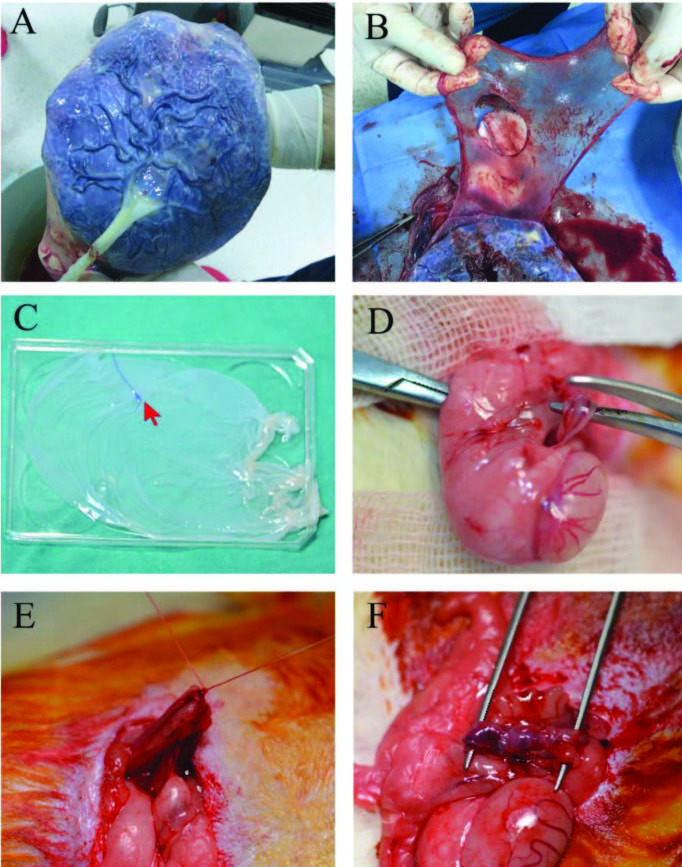
Obtaining human amniotic membrane and performing the experiment (A) Appearance of the amniotic membrane on the placenta. (B) Separation of the amniotic membrane from the placenta by blunt dissection. The aperture in the middle is the entry point of the umbilical cord. (C) After washing and cleansing, the amniotic membrane was stored in a sterile container. The inner side (the epithelial side) of the amniotic membrane was marked with a suture to distinguish the epithelial side from the chorionic side (red arrow). (D) Isolation and transection of the left vas deferens in the middle with scissors. (E) Anastomosis by performing vasovasostomy of transected vas deferens of rats in Group-III. (F) Wrapping the amniotic membrane on the anastomosis line in rats in Group-IV. The amniotic membrane is indicated with a black arrow.

HAM was prepared from the placenta of a pregnant woman who was delivered by elective cesarean section. She was seronegative for HIV, hepatitis B and syphilis in preoperative tests and had no history of premature rupture of membranes, endometritis or meconium ileus. HAM was separated from the chorionic membrane by blunt dissection and then washed four times in a sterile container with a large volume of saline to remove blood clots, mucus and foreign tissue (Figures 1A and 1B), as previously described by Ravishanker et al. [11]. After cleansing, the epithelial layer side was marked with a suture to distinguish it from the chorionicside (Figure 1C). HAM was stored for 24 h in 500 mL sterile saline containing 50 mg/mL penicillin, 50 mg/mL streptomycin, 100 mg/mL tobramycin and 2.5 mg/mL amphotericin B in a refrigerator at +4 °C (Figure 1C). It was used in the experiment after 24 h.

### 2.3. Performing of the experiment

The following procedures were performed sequentially in the rats other than those in Group-I:

1. Ketamine 50 mg/kg and xylazine 5 mg/kg were administered intraperitoneally for general anesthesia. A left inguinal incision was performed, and the left VD was found and transected in the middle with scissors (Figure 1D).

2. The skin of the rats in Group-II was sutured without any further procedures. In Group-III, end-to-end anastomosis was performed by vasovasostomy (Figure1E). In Group-IV, end-to-end anastomosis was performed by vasovasostomy, and a 2 × 1 cm piece of HAM was wrapped around the anastomosis line with the epithelial side inward. This HAM wrapping was secured with 7/0 polyglactin 910 surgical sutures (Pegelak, G0882, Dogsan, Trabzon, Turkey) (Figure 1F). 

3. Inguinal incisions were closed with 3/0 polypropylene sutures (Propilen, P4263, Dogsan, Trabzon, Turkey). Vasovasostomy was performed using 7/0 polyglactin 910 sutures.

4. All rats were sacrificed after 60 days. The left VD and testis were removed. Specimens were sent to the laboratory for histopathological examination. They were examined both macroscopically and histopathologically. Macroscopically, it was determined whether the anastomosis was retained or separated, whether there was spermatic granuloma (SG) and whether there was adherence to surrounding tissues. 

5. A 24G catheter was inserted into the proximal end of the VD, and patency was checked by passing methylene blue through it. This ascertained whether testicular atrophy was present.

VD patency of the rats was evaluated as no separation, separated in a granuloma, and separated. Luminal patency was evaluated as fully open, open but stenotic, and enclosed.

The rats were evaluated according to whether SG, adhesions to surrounding tissues and testicular atrophy were present (Table 2). Histopathologically, tissue samples were stained with hematoxylin-eosin and Masson’s trichrome stain and evaluated under a light microscope. It was evaluated whether fibrosis and stenosis were present in the anastomosis line. Then, the groups were compared statistically.

**Table 2:** Statistical analysis of the groups in terms of whether the vas deferens had separate ends, luminal patency, spermatic granuloma, adhesions to surrounding tissues and testicular atrophy.

Variables		Group I	Group II	Group III	Group IV	p*	p**	p***	p****
Separation of vas deferens	Intact	10	0	3	8	<0.001	0.03	<0.001	0.005
	Separated in granuloma	0	0	3	1				
	Separated	0	10	4	1				
Luminal patency	Fully open	10	0	3	7	<0.001	0.009	<0.001	0.013
	Open but stenotic	0	0	2	1				
	Enclosed	0	10	5	2				
Spermatic granuloma	None	10	3	4	5	0.01	0.66	0.37	0.65
	Present	0	7	6	5				
Adhesions to surrounding tissues	None	10	3	4	9	0.001	0.02	0.008	0.65
	Present	0	7	6	1				
Testicularatrophy	None	10	9	9	10	0.56	0.31	0.31	1.0
	Present	0	1	1	0				

* Kruskal–Wallis test applied; all groups were compared.

### 2.4. Statistical analysis

The data obtained were evaluated using SPSS version 21.0 software (IBM Corp., Armonk, NY, USA). Since all variables were categorical, the Kruskal–Wallis test was used when all four groups were compared. The Mann–Whitney U test and Bonferroni correction were used for pairwise comparisons. p < 0.05 was considered statistically significant for all variables.

## 3. Results

**Figure 2:**
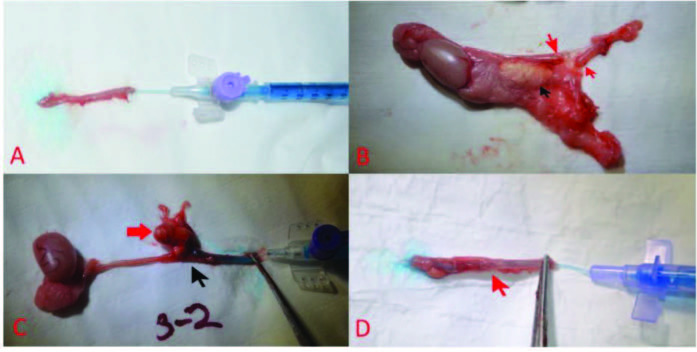
Macroscopic appearance of the left vas deferens after two months. (A) The vas deferens of the rats in the control group (Group-I) had a normal appearance, and the methyleneblue injected to test for luminal patency passed easily. (B) In Group-II, the vas deferens of all rats were completely separated. The separated tips are indicated by red arrows. Seven had spermatic granuloma. Spermatic granuloma is marked with a black arrow. (C) The ends of the vas deferens of three rats in Group-III were adjacent in the spermatic granuloma, but the lumen patency was closed, and there was stenosis or no passage of methylene blue. The anastomosis line is indicated by the black arrow, and granuloma is indicated by the red arrow. The anastomosis tips of four rats were separated. The anastomosis lines of three rats had a normal appearance. (D) In Group-IV, there was no stenosis in the anastomosis line in five subjects, and the methylene blue delivered from the proximal end passed easily. In four subjects, there was partial stenosis in the anastomosis line. The anastomosis line is indicated by the red arrow.

The VD in Group-I were normal both macroscopically and microscopically (Figure 2A). In Group-II, the VD of all rats (100%) were observed to be separated. When methylene blue was injected to assess patency, it was seen to discharge from the separated ends (Figure 2B). In Group-III, the anastomosis was intact in three rats (30%), it was completely separated in four rats (40%), and the tips of the VD were lying side-by-side in SG in three rats (30%) (Figure 2C). In the three macroscopically intact rats, lumen patency was open and methylene blue passed through. In the next four rats, the VD was completely separated; when methylene blue was injected, it spread from the split ends into the environment. There was no passage in one of the three rats ending in SG, while the other two had narrowing in the lumen due to stenosis. In Group-IV, the anastomosis was intact in eight of the ten rats (80%), and the ends of the anastomosis were separated and not intact in one rat (10%). In the other rat (10%), the tips of the anastomosis were lying side-by-side in SG. Seven of the eight rats (70%) with intact anastomosis showed a normal passage and no stenosis based on the methylene blue test (Figure 2D), while partial stenosis was observed in one rat (10%). No lumen passage was observed in two rats (20%): the one with the separated anastomosis ends and the one with the ends lying side-by-side in SG. 

The anastomosis resulted in a much better outcomes in the HAM group compared to the non-HAM group. The remaining intact rate of anastomosis in Group-IV was found to be higher than the intact rate in Group-III. The rate of stenosis formation was lower in Group-IV (Table 2). 

The statistical analysis of the groups is shown in Table 2. When all groups were compared, there were significant differences between the groups in terms of separation of the anastomosis (p < 0.001). In binary comparisons, there was less separation in the HAM group than in the non-HAM group, and this difference was statistically significant (p = 0.03). There were also significant differences in binary comparisons between Group-II and the other groups (Group-III and Group-IV) (p < 0.001 and p = 0.005, respectively) (Table 2).

When all groups were compared in terms of luminal patency, there was a significant difference between groups (p < 0.001). In binary comparisons, significant differences were found between Group-III and Group-IV (p = 0.009). There were also significant differences in binary comparisons between Group-II and Group-III/Group-IV (p < 0.001 and p= 0.013, respectively) (Table 2). 

SG was formed at different rates in all three noncontrol groups, but at a lower rate in Group-IV. It was observed in seven rats (70%) in Group-II, in six rats (60%) in Group-III and in five rats (50%) in Group-IV. There was a significant difference between the control group and the other groups (p < 0.001). There was no significant difference between Group-III and Group-IV in binary comparisons (p = 0.066). There were also no significant differences in binary comparisons between Group-II and Group-III/Group-IV (p < 0.37 and p = 0.65, respectively) (Table 2). 

There was considerable attachment between the VD and the surrounding tissues, especially at the incision lines, in seven rats (70%) in Group-II, six rats (60%) in Group-III and only one rat (10%) in Group-IV (Table 2). There was much less adhesion to the surrounding tissues in Group-IV than in the other groups. Comparing all groups in terms of adhesion to surrounding tissues, a significant difference was found between groups (p = 0.001). In binary comparisons, significant differences were found between Group-IIIand Group-IV (p = 0.02). Significant differences were also observed in the binary comparisons between Group-II and Group-IV (p = 0.008), but there was no difference between Group-II and Group-III (p = 0.65) (Table 2). 

Macroscopic assessment revealed left testicular atrophy in two rats: one (10%) in Group-II and one (10%) in Group-III. Comparing all groups, there was no significant difference between groups (p = 0.56). There was no difference between groups in binary comparisons (Table 2). 

**Figure 3:**
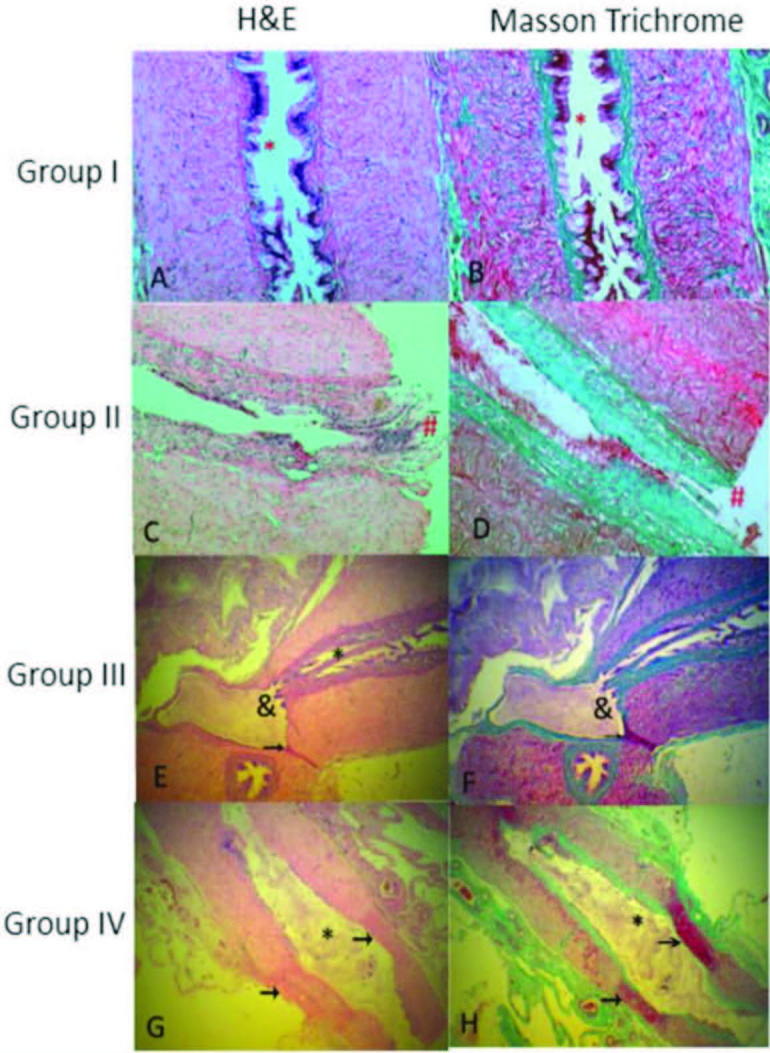
Appearance of the vas deferens of rats under light microscopy. In the first column (A, C, E, G), hematoxylin-eosin stained sections are shown. In the second column (B, D, F, H), Masson’s trichrome stained sections are shown. A and B) Vas deferens of Group-I (control group); lumen openings (*) are seen. C and D) Appearance of the vas deferens of subjects in Group-II; separate ends are indicated by (#). E and F) The ends of the vas deferens of the rats in Group-III were adjacent in the spermatic granuloma, but there was no lumen patency, and there was no passage or they were stenotic (&). There was no connection between the two ends. Recovery with scar on the incision line is indicated by the arrow. G and H) The vas deferens of Group-IV subjects showed clear luminal healing, good lumen clearance (*) and incision healing with scar (arrows indicate scarring on both walls). G and H) In Group-IV, the vas deferens had recovered better, resulting in lumen patency (*) and less scar on the incision line (scar on both walls indicated by arrows).

Figure 3 shows the images of the VD from each group with hematoxylin-eosin and Masson’s trichrome staining under light microscopy. In Group-I, normal VD was observed (Figures 3A and 3B). In Group-II, the tips of the VD were separated (Figures 3C and 3D). In Group-III, stenosis and fibrosis as well as SG were observed at the anastomosis line (Figures 3E and 3F). On the other hand, less fibrosis and scarring were observed in Group-IV, resulting in better healing of the VD with less stenosis (Figures 3G and 3H).

## 4. Discussion

Our results showed that VD treated with HAM had less scar formation and better luminal patency than those where HAM was not used in the anastomosis line. Also, VD was less adherent to the surrounding tissues in these rats. Although there was no statistically significant difference, it was observed that less SG developed in HAM used rats.

HAM is a biological membrane that accelerates wound healing, owing to its antiinflammatory, antibacterial, antiviral and angiogenic properties. It also enhances epithelialization, slows apoptosis and has low immunogenicity. It was first used by Davis et al. in 1910 for skin transplantation. Shortly afterwards, in 1913, it was used by Stern and Sabella with burn patients [6]. In 1940, de Rotth began to use it in ophthalmology for the first time in patients with symblepharon. Since then, it has become widely used in the treatment of corneal ulcers and injuries [12]. It is used in the treatment of Stevens–Johnson syndrome and numerous diseases in plastic surgery. It has also been used in dentistry since the end of the twentieth century. Experimental studies in rats have shown that HAM improves the healing of spinal cord injuries [13]. Serena et al. demonstrated that HAM accelerates healing of chronic diabetic foot ulcers [14]. HAM has also been found to be useful in tympanoplasty, arthroplasty and reconstruction of the bladder and vagina [15]. A recent study has shown that it is also useful in hypospadias repair [16].

HAECs are pluripotent cells because they originate from the epiblast. They also display properties that are similar to MSCs. They can therefore be expected to possess the desirable clinical benefits of MSCs. HAECs also have immune-privileged properties [17]. They express the nonpolymorphic, nonclassical human leukocyte antigen (HLA-G), but they do not express the polymorphic antigens HLA-A, HLA-B (Class IA) and HLA-DR (Class II) [18]. Therefore, HAM has low immunogenicity, and no rejection occurs after using it.

Recently, HAM has been used in tissue engineering and regenerative medicine because of the pluripotent, self-renewal properties of the cells it contains [6]. Also, its skeletal structure is suitable for use as a scaffold, and it has many growth factors and cytokines used in tissue engineering [19]. These unique features and easy availability make it an ideal candidate for use in numerous clinical situations; HAM has great potential, both in the treatment of wound healing and in the field of tissue engineering [20]. It is safe, cheap and easy to obtain when collected under suitable conditions. 

HAM accelerates reepithelialization and wound healing, owing to the abovementioned antimicrobial, antiinflammatory, angiogenic and low immunogenic properties; it also provides scarless (or reduced scar) wound healing through its antifibrotic effect [21]. 

Allograft and xenogenic materials implanted in an organism cause a foreign body reaction and initiate an inflammatory reaction. Inflammatory cytokines released from the inflammatory cells collected in the area also cause migration of fibroblasts and result in fibrosis. Fibroblasts are activated by transforming growth factor-β (TGF-β). HAM downregulates TGF-β and its receptor expression by fibroblasts and reduces the risk of fibrosis. The superfamily of TGF-β has three isoforms (TGF-β1, TGF-β2 and TGF-β3 isoforms), but in spite of their structural similarity, they have different and sometimes inverse functions [22]. TGF-β1 and TGF-β2 act as chemoattractants for immune cells and activate the inflammatory response; this leads to the proliferation of fibroblasts. They also increase the synthesis of the extracellular matrix. As a result, collagen deposition is increased. These cytokines thereby accelerate fibrosis and eventually the formation of a scar [23]. Contrarily, TGF-β3 prevents migration and proliferation of inflammatory cells by antagonizing TGF-β1, and it acts as an antifibrotic cytokine. It has been shown that HAM contains TGF-β3 cytokine produced by HAECs; the secretion of TGF-β3 makes amnion a perfect source when inhibition of scarring is desired [24]. HAM can modulate wound healing by promoting tissue reconstruction instead of scar tissue formation [25]. The results of this study also showed that HAM reduced fibrosis and scar formation histopathologically (Figures 2 and 3). 

SG is thought to develop as a result of a chronic immune response to extravasated sperm caused by trauma, surgery or infection [26]. When the blood-epididymal barrier is disrupted, the host immune system perceives the extravasated sperm as foreign bodies and tries to isolate them by surrounding them with macrophages and connective tissue [27]. Group-IV had the lowest granuloma rate (50%), while Group-II had the highest rate (70%). This was attributed to less leakage and suppression of the antiinflammatory response, possibly due to the surrounding HAM creating a physical barrier to the anastomosis line; however, there was no significant difference between Group-III and Group-IV. 

In the group using HAM, the observation of less adhesion to surrounding tissues was attributed to both the physical barrier effect of HAM and the antiinflammatory effect of the mechanism described above.

This study has several limitations. First, we could not investigate the immunological parameters that could affect the injury healing. Second, this is an animal study, and all of the findings should not be projected to human beings.

In conclusion, it was seen that when repairing IVDI, wrapping HAM over the vasovasostomy line provided better healing of the anastomosis and less stenosis. The results of the study suggest that HAM can be used for repairs in the treatment of IVDIs, which often occur during inguinal region operations in children, to secure vasovasostomy anastomosis. This is the first study to investigate the use of HAM in IVDI treatment. This study therefore proposes the use of HAM in an area where it has not been used before. It is necessary to carry out additional clinical studies on humans to further evaluate this novel use of HAM.
